# In silico predicted epitopes from the COOH-terminal extension of cysteine proteinase B inducing distinct immune responses during *Leishmania (Leishmania) amazonensis *experimental murine infection

**DOI:** 10.1186/1471-2172-12-44

**Published:** 2011-08-08

**Authors:** Bernardo AS Pereira, Franklin S Silva, Karina M Rebello, Marcel Marín-Villa, Yara M Traub-Cseko, Thereza CB Andrade, Álvaro L Bertho, Ernesto R Caffarena, Carlos R Alves

**Affiliations:** 1 Laboratório de Biologia Molecular e Doenças Endêmicas, IOC,Fiocruz, Avenida Brasil, 4365 Manguinhos Pavilhão Leônidas Deane - sl. 209 CEP: 21040-900 Rio de Janeiro, RJ, Brasil; 2 Laboratório de Biologia Molecular de Parasitas e Vetores, IOC, Fiocruz, Rio de Janeiro, RJ, Brasil; 3 Laboratório de Imunologia Clínica, IOC, Fiocruz, Rio de Janeiro, RJ, Brasil; 4 Laboratório de Imunoparasitologia, IOC, Fiocruz, Rio de Janeiro, RJ, Brasil; 5 Programa de Computação Científica, Presidência, Fiocruz, Rio de Janeiro, RJ, Brasil

## Abstract

**Background:**

*Leishmania *parasites have been reported to interfere and even subvert their host immune responses to enhance their chances of survival and proliferation. Experimental *Leishmania *infection in mice has been widely used in the identification of specific parasite virulence factors involved in the interaction with the host immune system. Cysteine-proteinase B (CPB) is an important virulence factor in parasites from the *Leishmania (Leishmania) mexicana *complex: it inhibits lymphocytes Th1 and/or promotes Th2 responses either through proteolytic activity or through epitopes derived from its COOH-terminal extension. In the present study we analyzed the effects of *Leishmania (Leishmania) amazonensis *CPB COOH-terminal extension-derived peptides on cell cultures from murine strains with distinct levels of susceptibility to infection: BALB/c, highly susceptible, and CBA, mildly resistant.

**Results:**

Predicted epitopes, obtained by *in silico *mapping, displayed the ability to induce cell proliferation and expression of cytokines related to Th1 and Th2 responses. Furthermore, we applied *in silico *simulations to investigate how the MHC/epitopes interactions could be related to the immunomodulatory effects on cytokines, finding evidence that specific interaction patterns can be related to *in vitro *activities.

**Conclusions:**

Based on our results, we consider that some peptides from the CPB COOH-terminal extension may influence host immune responses in the murine infection, thus helping *Leishmania *survival.

## Background

Leishmaniasis, a vector-borne parasitic infection, is caused by protozoans of the genus *Leishmania*, which present a worldwide distribution. This disease is characterized by its diversity and complexity, presenting a wide spectrum of clinical forms in humans, ranging from self-healing skin lesions to fatal visceral leishmaniasis, depending on parasite species and host factors [1,2].

In Brazil, both clinical forms are present and, currently, leishmaniasis can be classified as a disease in expansion and without effective control [3]. One important etiological agent of human leishmaniasis in Brazil is *Leishmania (Leishmania) amazonensis*, which causes a wide spectrum of clinical diseases [4]. Interestingly, recent findings indicate that the geographical distribution of *L. (L.) amazonensis *is increasing, accounting for unusual clinical presentations in new transmission areas, specifically Rio de Janeiro State [5].

Amastigote forms of *Leishmania *are intracellular parasites, inhabiting preferentially cells of the mononuclear phagocyte system, and present a series of adaptive peculiarities in this phase of the biological cycle. These adaptations allow the parasites to escape or interfere with the host immune responses and, consequently, maintain the infection. The host immunological profiles during *Leishmania *infections are widely studied using the murine model, as distinct leishmaniasis clinical manifestations can be reproduced in inbred mice strains [reviewed in [6]].

In the murine *Leishmania (Leishmania) major *infection model it has been established that a T-helper cell type 1 (Th1) immune response is effective against the parasites, while a Th2 response may lead to disease exacerbation. Thus, in this system, the outcome of infection is determined by the balance between these two types of response [7]. However, this dichotomy is not so clearly observed in murine infections caused by the *Leishmania (Leishmania) mexicana *complex species, as *L. (L.) amazonensis*, where the susceptibility of the host to the infection seems to be associated with an insufficient Th1 response rather than a dominant Th2 response [8]. The distinct levels of susceptibility to infection observed in different mouse strains, e.g. BALB/c mice being highly susceptible and CBA mice having some degree of resistance, may be due to differences in a mixed Th responses balance rather than determined by a single dominant response. Additionally, regulatory cytokines, as IL-10, might influence the outcome of the infection in a manner not directly linked to the Th1/Th2 dichotomy [9].

In this context, cysteine proteinases (CPs) (E.C. 3.4.22) are virulence factors for *Leishmania *with immunoregulatory properties during infection [10]. Most of the studies on *Leishmania *CPs are centered in a few enzymes, denominated CPA, CPB and CPC, all of which are similar to papain and belong to the same group of CPs (designated Clan CA, Family C1). However, a detailed analysis of the *L. (L.) major *genome database revealed the existence of genes predicted to codify for a total of 56 CPs, subdivided into 4 Clans and 13 Families [10].

The current focus of our studies is CPB, a cathepsin L-like enzyme [11], which presents exclusive characteristics when compared to other CPs: an extension of approximately 100 aa in its COOH-terminal region and the presence of multicopies organized as tandem array in the parasite genome [10]. CPB from *L. (L.) mexicana *and *L. (L.) amazonensis *have been shown to have a role on the Th1/Th2 responses balance by cleaving type II major histocompatibility complex (MHC) molecules in the host parasitophorous vacuole [12], cleaving the interleukin (IL)-2 and immunoglobulin (Ig) E receptors, inducing IL-4 expression [13], inhibiting IL-12 production in macrophages and dentritic cells [14] and by cleaving host nuclear factor kappa B (NF-κB) [15]. Considering that during the processing of pre-CPB to its mature form the COOH-terminal extension is processed [16], secreted [17] and can be observed within host cells [18], this released fragment may also play a role in the host-parasite interaction [19].

Our group previously published an *in silico *study, using the analysis of physicochemical characteristics as parameter for T cell epitopes prediction, that allowed the identification of three peptides from *L. (L.) mexicana *CPB COOH-terminal extension. In that study, three peptides with electric charges, hydrophobicity and isoelectric points compatible with the binding to MHC molecules were selected [20].

These peptides were chemically synthesized and used *in vivo *and *in vitro *studies using BALB/c and CBA mice. One of these peptides caused an exacerbating effect on BALB/c mice lesions, when used in vaccination assays. Moreover, lymphoproliferative assays indicated that CD8^+ ^T cells proliferated more intensely than CD4^+ ^T cells in the presence of that same peptide. When cytokine levels were assessed, interferon-γ (IFN-γ), a Th1 response-related cytokine, was detected only in the supernatant of CBA lymph node cell culture, while IL-4, a Th2 response-related cytokine, was detected in the supernatant of cell culture from both lineages. Additionally, nitric oxide (NO) was detected only in the supernatant of CBA cell cultures [21].

Such data corroborated the potential of CPB as an inducer of a Th2 immune response, showing that peptides derived from this enzyme may be influential co-stimulatory signals in T-cell polarization during leishmaniasis, able to interfere in the balance of the Th immune responses.

In the present study, we searched further for immunomodulatory peptides derived from the COOH-terminal extension of *L. (L.) amazonensis *CPB. This was achieved by using current online algorithms to directly define peptides able to bind to MHC molecules and by using *in vitro *assays to assess the effect of the various peptides on mouse strains with distinct levels of susceptibility to *L. (L.) amazonensis *infection.

## Results

### Sequencing of the *L. (L.) amazonensis *CPB COOH-terminal extension

**Figure 1:**
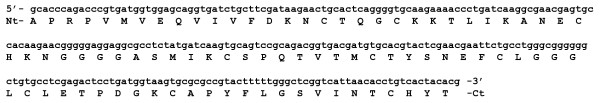
**Nucleotide (above) and amino acid (below) sequences of the COOH-terminal extension from *L. (L.) amazonensis *CPB**. Sequences are informed as 5' → 3' and NH_3_-terminal (Nt) → COOH-terminal (Ct), respectively.

*L. (L.) amazonensis *CPB COOH-terminal extension encoding genome sequence was amplified and cloned using primers previously produced for an analogous region of *L. (L.) pifanoi *CPB. After determination of the DNA sequence, it was used as basis for deducing amino acids sequence of the COOH-terminal extension (Figure 1).

This sequence presented up to 93% of identity with sequences of CPs from other *Leishmania *species belonging to the *L. (L.) mexicana *complex and up to 68% with species from other complexes, as indicated by a protein BLAST analysis using the compositional matrix adjust method.

### *In silico *prediction of T cell epitopes present at the COOH-terminal extension of *L. (L.) amazonensis *CPB

The deduced amino acids sequence for the COOH-terminal extension of *L. (L.) amazonensis *CPB was analyzed by online algorithms to predict proteasome cleavage (NetChop and PAProC) and MHC-binding peptides (SYFPEITHI). Only peptides commonly predicted by the three algorithms used were considered for this study (Table 1). These predicted epitopes were then synthesized for *in vitro *analyses. All synthetic peptides were tested for endotoxin presence and presented values below the assay threshold (data not shown).

**Table 1:** Putative T lymphocytes epitopes from the COOH-terminal extension from *L. (L.) amazonensis *CPB

Epitope	Amino acids sequence	Number of amino acids	Binds to H-2 haplotype (predicted)	SYFPEITHI score value
P1	KNGGGGASMI	10	D	13

P2	NGGGGASMI	9	d and k	15/13

P3	GGGGASMI	8	K	13

P4	MCTYSNEFCL	10	D	10

P5	CTYSNEFCL	9	D	12

P6	FCLGGGLCL	9	D	17

P7	CAPYFLGSVI	10	D	11

P8	APYFLGSVI	9	d and k	13/13

P9	PYFLGSVI	8	K	13

Each synthetic peptide was assayed only with the mouse strain that presented the haplotype to which it showed binding properties in SYFPEITHI analysis. Thus, P1, P4, P5, P6 and P7 were assayed only with BALB/c mice, P3 and P9 were assayed only with CBA mice, while P2 and P8 were assayed with both mice strains.

### T lymphocytes proliferative responses induced by synthetic peptides

**Figure 2:**
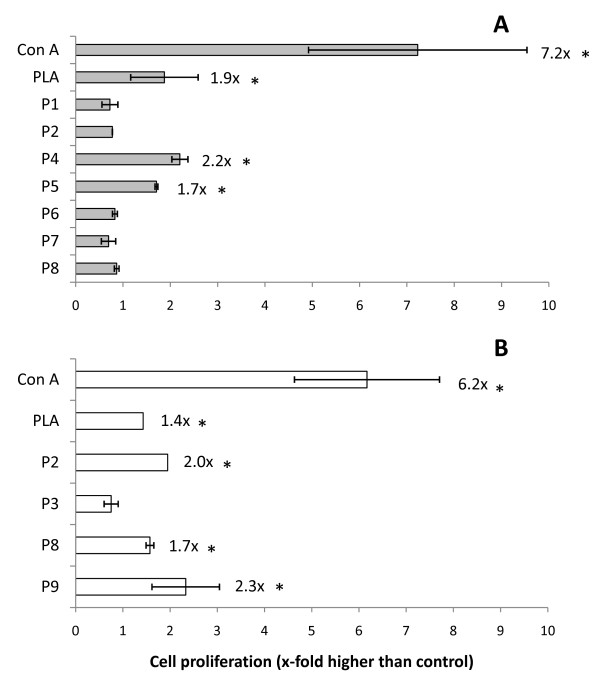
**Blastogenesis of murine lymph node cells induced by synthetic peptides derived from the COOH-terminal extension of *L. (L.) amazonensis *CPB**. Cell cultures from 15 infected BALB/c (A) or CBA (B) mice were incubated for 3 days in 96-well plates with synthetic peptides (30 μg/mL), PLA (30 μg/mL) or Con A (2 μg/mL), coincubated with [^3^H]-timidine (0.1 μCi/well) in the final 16 h of culture and incorporated radioactivity (indicative of cell proliferation) was measured in a beta-scintillation counter. Results are from a representative assay from at least three independent repeats. *Statistically distinct from control (*p *< 0.05).

Assays carried out with BALB/c cells showed that peptides P4 and P5 were able to induce a low level of cell proliferation when co-incubated with lymph node cells (2.2-fold and 1.7-fold increase in cell number, respectively) (Figure 2A). As for CBA mice cells, peptides P2, P8 and P9 also showed activity (2-fold, 1.7-fold and 2.3-fold increase in cell number, respectively) (Figure 2B). Positive blastogenesis control with Con A showed a 7.2-fold and 6.2-fold increase in cell numbers for BALB/c and CBA cell cultures, respectively. Specific blastogenic response control with Particulate *Leishmania *antigen (PLA) also showed a slight proliferation (1.9-fold and 1.4-fold increase in cell number for BALB/c and CBA, respectively).

### Influence of the predicted epitopes on lymph node cells phenotype

**Figure 3:**
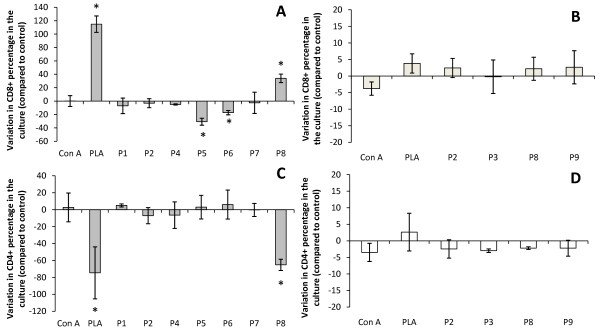
**Percentages of CD8+ (A and B) and CD4+ cells (C and D) in murine lymph node cell cultures after incubation with synthetic peptides derived from the COOH-terminal extension of *L. (L.) amazonensis *CPB**. Cell cultures from 15 infected BALB/c (A and C) or CBA (B and D) mice were incubated for 3 days in 24-well plates with synthetic peptides (30 μg/mL), PLA (30 μg/mL) or Con A (2 μg/mL), stained with specific CD4 and CD8 antibodies conjugated to fluorophores and submitted to analysis by flow cytometry. Results are from a representative assay from at least three independent repeats. *Statistically distinct from control (*p *< 0.05).

BALB/c lymph node cell cultures incubated with peptides P5 and P6 showed a decrease in the percentage of CD8^+ ^T cells (31% and 17% decrease when compared to control cultures, respectively). In contrast, peptide P8 was able to increase the percentage of BALB/c CD8^+ ^T cells present in the cultures by 34% (Figure 3A). As for the percentage of CD4+ T cells in the cultures, P8 was able to reduce the number of this cell type in the BALB/c lymph node cells by 65% (Figure 3D). None of the tested peptides was able to significantly alter CD8^+ ^or CD4^+ ^percentages in lymph node cell cultures from CBA mice.

P8 showed a strong influence over both CD4^+ ^and CD8^+ ^in BALB/c cells, being able to alter the proportions between these two cell types in the culture. While the CD8+:CD4+ proportion in control cultures was of 0.5, P8-incubated cultures presented a proportion of 1.8. Peptides P5 and P6 also altered the proportion between these two cell types, but as they influenced only CD8^+ ^cell numbers these alterations were less significant, establishing a CD8^+^:CD4^+ ^proportion of 0.3, after incubation with peptides.

Blastogenesis positive control (incubated with Con A) did not present any variations on CD8^+^:CD4^+ ^proportions, while cultures with PLA showed results comparable to what was observed for P8: CD8^+ ^cells percentage increased by 115%, CD4^+ ^cells percentage decreased by 75% and CD8^+^:CD4^+ ^proportion of 3.5.

### Production of cytokines and nitric oxide by cells in culture incubated with predicted epitopes

The levels of expression of cytokines related to Th1 and Th2 responses were analyzed in the supernatants of lymph node cell cultures co-incubated with the synthetic peptides. Also, the levels of IL-10 expression, a cytokine currently known to act in regulation of immune responses, were tested. In parallel, the NO levels in these supernatants were also analyzed. The basic values of all the assays (the mean values obtained for negative control cell cultures) were subtracted from the values obtained from all the samples and positive controls. The cytokine profiles in these cell cultures can be related to the capacity of the infected mice to control *Leishmania *infection, thus these parameters were analyzed in cell cultures from BALB/c (Table 2) and CBA (Table 3) mice. One Th2-related cytokine (IL-4), two Th1-related cytokines (IL-12 and IFN-γ) and one regulatory cytokine (IL-10) were tested.

**Table 2:** Cytokines levels in lymphnode cell cultures from infected BALB/c mice after coincubation with synthetic peptides

pg/mL					μM
	
	IL-4	IL-10	IL-12	IFN-γ	NO
Con A	979.69 ± 3.79	590.30 ± 77.20	32.16 ± 5,21	872.33 ± 17.12	7.78 ± 3.49
PLA	37.82 ± 5.67	188.30 ± 34.88	12.02 ± 11,77	474.92 ± 92.24	0.84 ± 0.13
P1	ND	ND	75.00 ± 34.65	ND	ND
P2	ND	ND	ND	15.88 ± 3.19	ND
P4	ND	10.97 ± 2.47	ND	49.25 ± 20.10	ND
P5	ND	ND	ND	ND	ND
P6	ND	2.97 ± 0.82	ND	ND	ND
P7	ND	ND	ND	ND	ND
P8	ND	ND	ND	ND	ND

Results presented are from a representative assay from three independent repeats using lymph nodes from 15 infected mice. Results are presented as mean ± standard deviation values. ND - below the threshold established by control group.

**Table 3:** Cytokines levels in lymphnode cell cultures from infected CBA mice after coincubation with synthetic peptides

pg/mL					μM
	
	IL-4	IL-10	IL-12	IFN-γ	NO
Con A	226.84 ± 9.53	338.10 ± 11.49	26.85 ± 11.94	939.48 ± 0.00	ND
PLA	33.67 ± 4.82	68.57 ± 6,88	ND	87.06 ± 12,23	1.39 ± 0.96
P2	ND	ND	18.37 ± 11.58	ND	ND
P3	ND	ND	16.44 ± 3.25	ND	ND
P8	ND	27.46 ± 0.74	ND	ND	ND
P9	ND	21.03 ± 0.16	8.02 ± 3.85	ND	ND

Results presented are from a representative assay from three independent repeats using lymph nodes from 15 infected mice. Results are presented as mean ± standard deviation values. ND - below the threshold established by control group.

In BALB/c cell cultures, peptides P1 and P2 promoted the expression of Th1-related cytokines (IL-12 and IFN-γ, respectively), while P6 promoted the expression of IL-10. The peptide P4 was able to induce cytokines from distinct Th responses in these cell cultures (IL-10 and IFN-γ). Con A and PLA controls were able to induce the expression of all tested cytokines.

Assays with CBA cells evidenced the capacity of peptides P2 and P3 to induce the expression of a Th1-related cytokine (IL-12), whereas peptides P8 induced the expression of a regulatory cytokine (IL-10). As observed in BALB/c cell cultures, one of the tested peptides (P9) was able to promote the expression of cytokines from multiple Th responses (IL-10 and IL-12). In CBA cell cultures, Con A displayed an activity similar to the pattern observed with BALB/c cells, but PLA was unable to promote IL-12 expression, although inducing the expression of all other tested cytokines.

None of the tested peptides was able to induce NO production in cell cultures from the tested murine strains, although, in BALB/c cultures, Con A and PLA enhanced NO levels in supernatants. In CBA cell cultures, only PLA enhanced NO levels in the supernatant.

### H-2/peptides complexes interactions predicted by molecular docking assays

**Figure 4:**
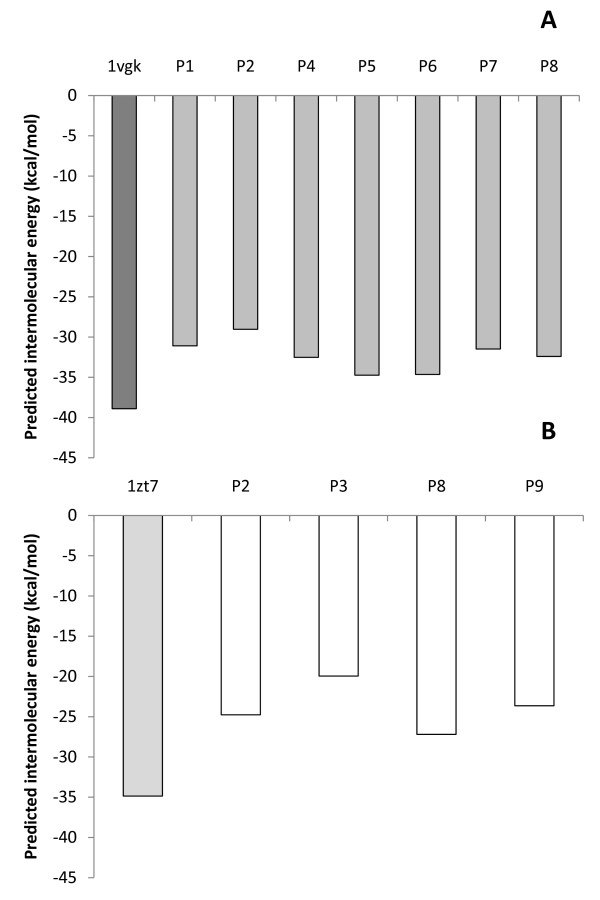
**Predicted intermolecular energy (kcal/mol) of the complexes H2/epitope by the *in silico *docking assays**. Putative epitopes P1, P2, P4, P5, P6, P7 and P8 were assayed with H-2K^d ^- BALB/c mice (A), while P2, P3, P8 and P9 were assayed with H-2K^k ^- CBA mice (B). Control assays were performed by redocking the original cocrystallized epitopes to their respective H-2 haplotype (PDB codes: 1vgk and 1zt7).

**Figure 5:**
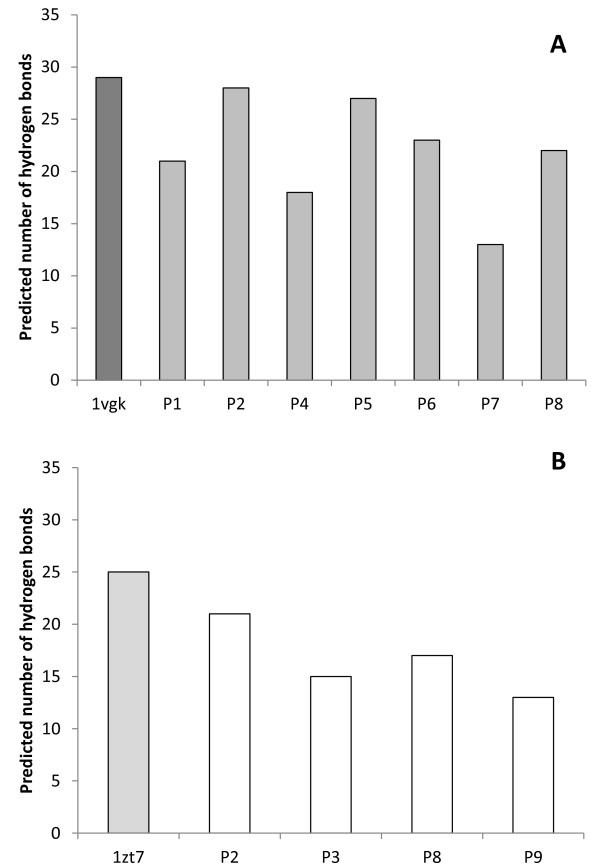
**Predicted number of van der Waals contacts of the complexes H2/epitopes by the *in silico *docking assays**. Putative epitopes P1, P2, P4, P5, P6, P7 and P8 were assayed with H-2K^d ^- BALB/c mice (A), while P2, P3, P8 and P9 were assayed with H-2K^k ^- CBA mice (B). Control assays were performed by redocking the original cocrystallized epitopes to their respective H-2 haplotype (PDB codes: 1vgk and 1zt7).

**Figure 6:**
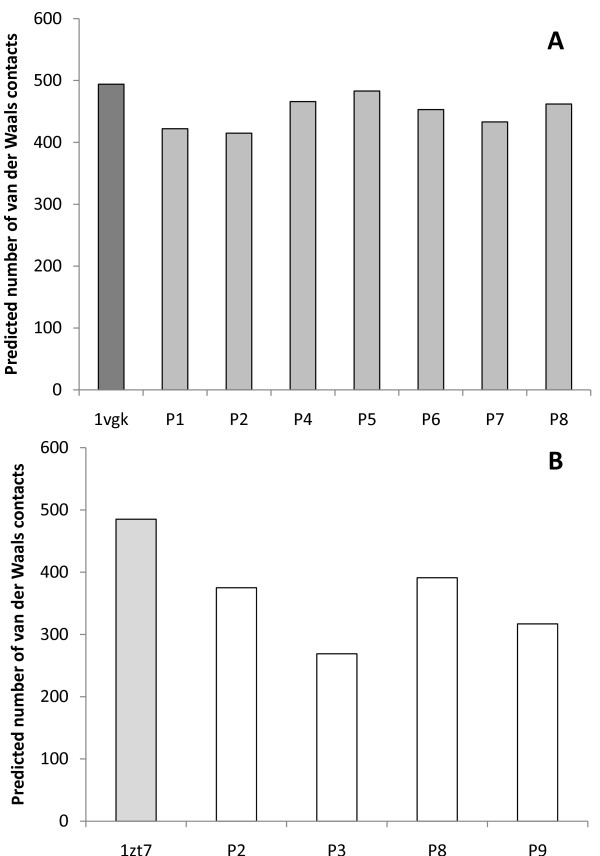
**Predicted number of hydrogen bonds of the complexes H-2/epitope by the *in silico *docking assays**. Putative epitopes P1, P2, P4, P5, P6, P7 and P8 were assayed with H-2K^d ^- BALB/c mice (A), while P2, P3, P8 and P9 were assayed with H-2K^k ^- CBA mice (B). Control assays were performed by redocking the original cocrystallized epitopes to their respective H-2 haplotype (PDB codes: 1vgk and 1zt7).

The number of hydrogen bonds and Van der Waals contacts, along with the intermolecular energies of the complexes were calculated using virtual models by molecular docking assays (Figures 4, 5 and 6). Such data are important to verify if binding of peptides to H-2 cleft, according to molecular docking analysis, is feasible.

Redocking assays of the peptides, originally present in the cleft, were used as control. For H-2K^k^/1zt7 complex, redocking assays presented 18% of success rate (considering an acceptable RMSD variation of up to 1.5%), while H-2K^d^/1vgk assays presented 100% of success under the same parameter. The significantly lower success rate observed for H-2K^k^/1zt7 complex redocking can be related to the higher number of side chain dihedral angles of the ligand (28 points of torsion), whereas peptide 1vgk presents 17 points of torsion. Despite the apparent low redocking success rate for H-2K^k^/1zt7 complex, the best model obtained by the *in silico *assays presented an excellent match with the crystallized structure, thus proving adequate for the proposed analysis.

Molecular interaction data may also be helpful to understand how the H-2 molecules and ligands interact, when a qualitative/quantitative analysis is applied rather than a simply quantitative one, and the observed molecular interaction patterns can be related to the biological effects caused by the peptides.

The docking assays predicted that some amino acids from the H-2 cleft present key importance in the interaction of the MHC molecule with the epitope, evidenced through their capacity of forming a high number of hydrogen bonds and Van der Waals interactions. For the haplotype d (H-2K^d^) these apparently prevalent amino acids were: Gln 63, Arg 66, Trp 73, Arg 97, Thr 143, Trp 147, Tyr 155 and Tyr 159. Also, some amino acids seem to have importance only for Van der Waals interactions: Phe 99, Tyr 156 and Trp 167. For the H-2K^k ^haplotype (from CBA mice), the relevant amino acids for hydrogen bonds and Van der Waals interactions were: Asn 70, Asn 77, Tyr 99, Tyr 116, Trp 147 and Asn 156, whereas those in which Van der Waals interactions significantly contributed were: Tyr 7, Arg 97, Tyr 123, Arg 155 and Tyr 159.

In addition, the participation of some residues from the MHC cleft in the epitope binding was observed as exclusively or predominantly occurring for peptides able to induce the expression of a specific cytokine or a combination of cytokines in the cell cultures. Interactions were considered exclusive when formed only by peptides capable to induce a specific cytokine, while predominant interactions were formed by peptides able to induce a specific cytokine at least two times more frequently than by other peptides (Table 4).

**Table 4:** Predicted interactions in MHC/peptide complexes related to cytokines production in murine lymp hnode cell cultures

		IL-10		IL-12		IFN-γ		IL-10 + IL-12		IL-10 + IFN-γ	
**Specific MHC**	**Interactions**	**HB**	**VWC**	**HB**	**VWC**	**HB**	**VWC**	**HB**	**VWC**	**HB**	**VWC**

H-2K^d^	Exclusive	tyr 7	cys 164	*NP	*NP	*NP	phe 33	*NP	*NP	tyr 171	try 133 gly 149
	
	Predominant	try 73 tyr 159	*NP	try 147	*NP	gly 63	*NP	*NP	*NP	tyr 156	*NP

H-2K^k^	Exclusive	arg 155 H_2_O 373	*NP	*NP	gly 65 gly 69 phe 74	*NP	*NP	tyr 45	asp 152	*NP	*NP
	
	Predominant	*NP	*NP	asp 156	asn 70	*NP	*NP	*NP	*NP	*NP	*NP

Hydrogen bonds (HB) and Van der Waals contacts (VWC) were predicted by *in silico *Molecular Docking assays. Exclusive interactions (ExI) were predicted only between the specified murine strain H-2K molecule and the peptides that induce the discriminated cytokine production in lymph node cell cultures. Predominant interactions, despite not being exclusive, were predicted to occur at least twice more frequently for specific cytokine production-related peptides. *NP - none predicted.

## Discussion

Infection by *Leishmania *is a very complex process where both the host immune system and parasite virulence components interact and influence each other to respond in specific ways, thus determining the outcome of the infection. Some *L. (L.) amazonensis *factors are able to directly interfere with the host immune system and lead to a response that benefits parasite survival [12].

In the present study, we have aimed at defining a strategy that, combining *in silico *and *in vitro *assays, would help to identify peptides from *L. (L.) amazonensis *that present immunoregulatory properties. We based our present analysis on putative epitopes derived from the COOH-terminal extension from *L. (L.) amazonensis *CPB, as our group had previously studied, by other methodologies, epitopes from this same region [20,21]. In our previous reports, epitopes mapping was performed by analyzing structural and chemical information of amino acids sequences rather than by applying sequences to algorithms designed for the simulation of epitopes processing and MHC interaction.

We initially sequenced the COOH-terminal region from *L. (L.) amazonensis *CPB. The sequence obtained, although very similar to the sequence described for *L. (L.) mexicana*, presented some differences in its deduced amino acid sequence, thus indicating the possible existence of epitopes exclusive for *L. (L.) amazonensis*. These exclusive epitopes may be related to some particularities of the infection caused by this *Leishmania *species, perhaps contributing to the stimulus of sub-populations of CD4^+ ^T cells related to pathogenesis [22].

A previous study reported the prediction of murine MHC class I-binding epitopes from *L. (L.) major *proteins using online algorithms. Differences in the number of predicted epitopes between a susceptible and a resistant mice strain were observed [23]. However, no *in vitro *analysis of the predicted epitopes was carried out and the prediction strategy was focused on finding as many epitopes as possible, merging results from four algorithms.

In the present study, we used a different strategy: by selecting only peptides which were predicted by all algorithms used (MHC-binding prediction and proteasomal cleavage prediction algorithms), we simulated actual epitope processing and, thus, selected only epitopes that had a high possibility to exist during murine infection. Additionally, we performed *in vitro *assays with synthetic versions of our selected epitopes, to test how accurate *in silico *prediction would be in selecting epitopes with immune effects during experimental infection.

We used a total lysate extract from the parasite (PLA), as a specific positive control in our assays, since it contains many potential antigens. This control induced blastogenesis in cell cultures from infected mice, apparently favoring CD8+T cells proliferation and inducing the production of cytokines related to multiple Th immune responses. Such effects of PLA are compatible to what was observed previously for another parasites lysate extract [21] and may account for the differences observed between infections with *L. (L.) amazonensis *or *L. (L.) major *in mice: while a mixed Th response profile can be observed in murine infections by *L. (L.) amazonensis*, there is always a unique and clearly defined Th response present in infections by *L. (L.) major *(either Th1 or Th2, depending on the mouse strain) [24,25].

Based on these observations, we performed assays designed to understand if all potential epitopes (peptides) derived from the COOH-extension of CPB would induce a unique and well-characterized Th response or if there would be peptides stimulating simultaneously distinct Th responses. Our results indicated that the latter hypothesis is true. It is interesting to note that CD8+ T lymphocytes may also present profiles of cytokines expression in a manner similar to CD4+ T lymphocytes [26] and, so, may be the cell type responsible for the production of the cytokines detected in the cell cultures. But none of the tested peptides was able to induce NO production in cell cultures of BALB/c or CBA mice.

This diversity of stimuli by the tested peptides points towards a possible survival strategy of the parasite: by overloading the host immune system with multiple immunomodulatory antigens, the parasite may induce it to elicit a series of inefficient responses and, thus, render it unable to properly control the infection. These inefficient responses would be translated as the mixed Th response observed in murine infection by *L. (L.) amazonensis *[24].

Contrary to what would be expected, the cells from CBA mice in culture did not produced IFN-γ when challenged with the synthetic epitopes. Although this fact may seem contradictory at first, because the expression of a Th1-related cytokine by a murine strain mildly resistant to *L. (L.) amazonensis *infection seems logic, a previous review article by Alexander & Bryson (2005)[7] already pointed out that, despite being useful to explain some of the features that lead to disease control/exacerbation in *Leishmania *infections, the Th1/Th2 dichotomy model is oversimplified. Thus, other factors, unrelated to these responses, may influence the outcome of infection. So, though we have tried to use the Th1/Th2 dichotomy as a guideline along this study, it was not unexpected to observe results that would not fit into the Th1/Th2 model, as the one indicated.

Also, in our study, we noted that stimulation for cytokines production seems to be unrelated to stimulus for proliferation. A possible explanation for this fact may be an inhibition of IL-2 or its receptor production in lymphocytes by the parasites: such strategy would limit the area of an immune response, allowing for dissemination of the infection. Deficiency to produce IL-2 was previously reported in patients with visceral leishmaniasis [27], while in mice infected by *L. (L.) amazonensis *such inhibition in the production of this cytokine was not observed [28]. Further investigation of IL-2 production and activity in the murine model of *Leishmania *infection is required to clarify this question.

*In silico *simulations of the interactions between MHC and the predicted epitopes were also performed. These simulations were helpful to indicate some major amino acid residues of the MHC cleft that act on epitope binding. More importantly, they indicated some patterns of MHC/epitope interaction that seem to be related to the stimulus to produce a specific cytokine. Further studies with increasing numbers of epitopes that stimulate specific cytokines in experimental murine infection with *Leishmania *are required, but our results provide a preview of a study methodology to understand immunomodulatory effects of this parasite.

We were able to confirm, by the data gathered in this study, the potential of the COOH-terminal extension from *L. (L.) amazonensis *CPB to influence the outcome of experimental murine infections by interfering, through its derived peptides, with the balance of Th cells, as we had previously proposed [21].

Taken in conjunction with our previous reports, our present results shed light over another specific aspect of the highly complex and fine-tuned network of interactions that regulates the outcome of *Leishmania *infection in mice. Also, we believe that, if applied to the study of other potential immunoregulatory molecules from the parasite, the methodological approach drafted here may be very helpful for a fast definition of epitopes derived from parasites proteins.

## Conclusions

The proposed methodology for epitope mapping of *Leishmania *proteins was shown to be very useful. Many distinct predicted epitopes from the COOH-terminal extension from *L. (L.) amazonesis *CPB were able to promote the expression of distinct cytokines in murine cell cultures, thus showing important immunomodulatory properties of this extension.

The use of molecular docking technique to analyze the interaction between peptides and MHC molecules in the murine model of experimental *Leishmania *infection showed evidences of a relation between the capacity of peptides to induce the expression of specific cytokines in the cell cultures and their interaction with key amino acids residues in the MHC cleft.

## Methods

### Cultivation of parasites

*L. (L.) amazonensis *MHOM/BR/77/LTB0016 strain promastigotes (obtained from Coleção de *Leishmania *do Instituto Oswaldo Cruz - CLIOC/IOC - Fiocruz) were grown in Schneider's medium (Sigma) supplemented with 10% fetal bovine serum (FBS - Gibco, Invitrogen, Brazil) for 4 days at 28°C. For experimental infections, parasites were washed 3× with 50 mM phosphate buffered saline (PBS) (pH 7.2) centrifuged at 2,000 × *g *for 10 min at 4°C, counted using a Neubauer chamber and suspended in PBS at a concentration of 2 × 10^7 ^cells/mL.

### Mice and experimental infection

Specific pathogen-free female 5-7 week old mice (strains BALB/c and CBA) were obtained from the animal care facility of Fiocruz (Centro de Criação de Animais de Laboratório - CECAL/Fiocruz). For experimental infection, each animal was inoculated subcutaneously with 1.0 × 10^6 ^log-phase promastigotes in PBS (50 μL) in the left hindpaw. All procedures using animals were approved by the Animal Ethics Committee of Fiocruz (Comissão de Ética no Uso de Animais - CEUA/Fiocruz; L-0006/07).

### Sequencing of *L. (L.) amazonensis *CP COOH-terminal extension

DNA was extracted from 1.0 × 10^8 ^log-phase promastigotes using the Wizard Genomic DNA purification kit (Promega, Madison, Wisconsin, USA). PCR was performed using specific sense and antisense primers for the COOH-terminal extension of *L. (L.) pifanoi *CP Lpcys2 (5'-GGATCCGCACCCAGACCCGTGATG-3' and 5'-AAGCTTCTACGTGTAGTGACAGGT-3') [16] and the PCR Core System (Promega). The PCR conditions were as follows: initial denaturation at 95°C for 1 min; 40 cycles of denaturation at 95°C for 1 min, annealing at 60°C for 30 sec and extension at 72°C for 1 min; and a final extension cycle (72°C, 5 min). All PCR assays were performed using an Eppendorf Mastercycler thermal cycler. The reaction products were analyzed by agarose (1%) gel electrophoresis stained with ethidium bromide. The target band was extracted using Wizard SV gel and PCR cleanup system (Promega) and inserted into pGEM-t Easy vector (Promega).

*Escherichia coli *competent cells were transformed by thermal shock method and grown overnight at 37°C on LB-agar plates supplemented with 100 μg/mL of ampicillin, 40 μg/mL of isopropyl β-D-1-thiogalactopyranoside and 40 μg/mL of 5-bromo-4-chloro-3-indolyl-beta-D-galacto-pyranoside. Recombinant bacteria were grown in liquid LB medium supplemented with 100 μg/mL ampicillin. Plasmids were prepared using STET solution (8% sacarose, 5% Tris-HCl pH 8.0, 50 μM EDTA pH 8.0, 5% Triton X-100), purified with polyethylene glycol 8000 and sequenced by PDTIS/Fiocruz DNA sequencing platform (using a 3730 DNA Analyser - Applied Biosystems).

The results and quality of the sequencing were assessed with BioEdit Sequence Alignment Editor Software and the sequences submitted to NCBI Basic Local Alignment Search Tool [BLAST] http://blast.ncbi.nlm.nih.gov/Blast.cgi, to check for similar sequences.

### T cell epitopes mapping strategy and peptide synthesis

Potential T cell epitopes derived from the COOH-terminal extension were selected *in silico*, using the combination of a T cell epitope binding algorithm (SYFPEITHI - http://www.syfpeithi.de) with proteasome cleavage prediction algorithms (NetChop- http://www.cbs.dtu.dk/services/NetChop; PAProC - http://www.paproc.de). In this study we considered only H2-K molecules for MHC-binding prediction. The cutoff used for SYFPEITHI algorithm results was a score value of 10, as it indicates the possibility of existence of at least one major anchor-amino acid at the analyzed peptide sequence. Only the peptides present in the intersection of the results from all used algorithms were elected for the study. Such strategy was used aiming to emulate more precisely the actual *in vivo *processing of peptides that bind to MHC molecules. The elected peptides were chemically synthesized by Creosalus (USA) with 95% of purity (checked by HPLC) and predicted MW-compatibility verified by ES-MS. Contamination of the peptides with endotoxin was verified using Limulus Amebocyte Lysate QCL-1000 kit (Lonza), according to manufacturer's instructions.

### *In vitro *Lymphoproliferative assays *in vitro*

Lymphoproliferative assays were performed with cells isolated from lesion draining lymph nodes of 15 mice (BALB/c or CBA), 10 weeks post-infection with *L. (L.) amazonensis*. After isolating and counting viable cells, these were resuspended in RPMI medium supplemented with 2% FBS and seeded into 96-wells flat bottom microplates (5 × 10^5 ^cels/well). Positive proliferation controls were carried out with concanavalin A (Con A - 2 μg/mL). PLA was used as specific proliferation control. This antigen was obtained by submitting *L. (L.) amazonensis *promastigotes to successive freeze-thaw cycles and centrifugation at 10,000 × *g*, 60 min and 4°C, after which the pellet was discarded. Both PLA and the synthetic peptides were assayed at a concentration of 30 μg/mL. The cell culture microplates were kept for 3 days at 37°C in an atmosphere of 5% CO_2_. In the final 16 h of incubation, prior to the collection of the cells onto filter paper, 0.1 μCi of [^3^H]-thymidine was added to each well. The amount of radioactivity incorporated by the cells was measured (in counts per minute - cpm) using scintillation liquid (toluene with 0.005% 1,4-bis(5-phenyloxazol-2-yl)benzene and 0.6% 2,5-Diphenyloxazole) and a beta scintillation counter (1600 CA, Packard Instrumental Co., USA). Samples were run in triplicate in each assay and at least three assays were performed for data analysis.

### Phenotypic analysis of the T cells and cytokines and NO production analysis

The phenotype of the peptide-responding cells from *L. (L.) amazonensis *infected mice (BALB/c and CBA) was determined using 2.5 × 10^6 ^lymph node cells/mL cultured in 24-well flat-bottom plates (Nunc) under the same conditions described above. The same positive and specific proliferation controls were used. After 3 days in culture, the supernatant was withdrawn and used in cytokine and NO_2_^- ^experiments (see below), while the cells were harvested, washed with PBS (500 × *g*, 10 min, 4°C) and resuspended in PBS containing 0.05% sodium azide and 2% FBS. Specific monoclonal antibodies PE-Cy5 Rat Anti-mouse CD4 and PE Rat anti-mouse CD8a (BD Pharmigen, USA) were added [1:100] and the cell suspensions were incubated for 30 min at 4°C. In the course of cultivation, cell cultures were observed by light microscopy to verify if their morphology presented any alteration that could suggest stress or death of cells. Finally, cells were washed with PBS (500 × *g*, 10 min, 4°C), resuspended at PBS with 1% paraformaldehyde and analyzed using an EPICS ALTRA flow cytometer (Beckman Coulter). For each sample, 10,000 lymphocytes were recorded in list mode, and registered on a logarithmic scale histogram. During data acquisition, the volume and inner complexity parameters of events were controlled to match those typical of murine lymphocytes. Analysis was performed with the EXPO32™ (Applied Cytometry Systems - Beckman Coulter, USA) and Summit 4.3 (Dako, USA) softwares.

The levels of cytokines (IL-12, IFN-γ,IL-4 and IL-10) in the supernatants of cell cultures obtained from immunophenotyping assays were assessed with Quantikyne colorimetric sandwich ELISA kits (R&D systems), according to manufacturer's instructions. The thresholds for cytokines detection indicated by the Quantikyne kits were: IL-12p70 - 2.5 pg/mL, IFN-γ - 2 pg/mL, IL-4 - 2 pg/mL and IL-10 - 4 pg/mL.

The nitrite (NO_2_^-^) concentration was used as a measure of nitric oxide (NO) production in the same supernatants using Griess reagent, as described previously [29]. Briefly, 100 μL of the supernatants was added to wells from 96-wells flat bottom plates and mixed with 100 μL of Griess reagent (2,5% phosphoric acid with 0.1% naphthylene diamine 2 HCl and 1% sulphanilamide). After incubation for 10 min at room temperature, absorbance for each well was defined at 540 nm. A standard curve using sodium nitrite (0-200 μM) was used to assess NO concentration in the supernatants. The threshold for NO_2_^- ^detection using Griess reagent is down to 10^-9^M.

Samples were run in triplicate at each assay and at least three assays were performed for data analysis.

### *In silico *assays (molecular docking)

For the docking simulations, we used murine class I MHC molecules as receptors. The structural data for H-2K^d ^(BALB/c) and H-2K^k ^(CBA) molecules were obtained from Protein Data Bank (PDB - codes: 1vgk and 1zt7, respectively). The H-2K^k ^molecule was co-crystallized with Influenza virus peptide ligands (FEANGNLI), while H-2k^d ^was obtained in complex with a synthetic peptide (SYVNTNMGL).

The original ligands, present at each H-2 molecules in their conformations retrieved from PDB file, were mutated using the Swiss-PDB viewer software to match the selected putative epitopes, keeping unchanged the crystallographic conformations of their backbones.

The H-2/epitope file complexes, for docking assays, were prepared using the GROMOS 96 force field [30]: initial structures were optimized by minimizing potential energy to adequate molecular crystallographic coordinates to the force field. Partial atomic charges and the protonation states were defined for each molecule, with the solvation parameters of each atom set considering the amino acids protonation state under pH 4.0 (zwitterionic state) [31]. The putative ligands were set to a semi-flexible state, where only side chain dihedral angles were allowed to change, keeping the receptor rigid, using AutoTors software [32]. Three highly conserved water molecules in the H-2 active sites, which are known to play important roles both in the maintenance of the active site structure and ligand binding [33], were kept in the H-2 structures for the docking assays.

The atomic cartesian coordinates of the crystallized H-2 molecules (receptors) were then obtained after removal of ligands from the *in silico *structure and, new files containing this information, along with their atomic force parameters (generated by the GROMOS 96 force field), were created for docking analyses. The AutoDock 3.5 software was used to perform the molecular docking assays. The grids matrix was calculated using a spacing of 0.375 Å. The center of each grid was defined using the central atom of the ligand backbone as reference.

For each putative epitope, 8 docking simulations were run each one generating 256 models of interaction, summing up a total of 2048 models defined for each epitope. These models were clustered in families using as parameter the root mean square deviation (RMSD) values between them: models were considered as members of the same family if their RMSD value differed in less than 3.5 Å. Families constituted of inadequate models, e.g. peptides interacting with the H-2 molecule outside its cleft, were dismissed.

Subsequently, the model presenting the best fit compared to original crystalized molecules files, defined by RMSD variance, was chosen for further analysis. In cases were multiple models presented the same RMSD variance values, the model with both the lower docked energy value and lower intermolecular energy values was selected.

The representative model of each family was assessed in conjunction with its related H-2 active site structure to identify the intermolecular interactions occurring: hydrogen bonds and Van der Waals contacts. The criteria to define the existence of each type of atoms interactions were: for hydrogen bonds - maximal distance of 2.5 Å between hydrogen atom and acceptor atoms, and, for Van der Waals contacts - maximal distance of 4.5 Å between non-charged atoms. Only the residues of each H-2 molecule that interacted at least 50% of the tested models were considered as relevant and conserved.

### Statistical analysis

Data from the *in vitro *assays were analyzed for statistical significance, using an unpaired Student's *t*-test, and considered significant when *p *< 0.05.

## Authors' contributions

BASP contributed in the design of the project, participated in all the assays and analysis performed for this study and drafted the manuscript. FSS participated in cell culture-related assays and helped executing and analyzing *in silico *assays. KMR participated in the execution of the *in silico *assays. MMV helped performing the assays to sequence *L. (L.) amazonensis *CP COOH-terminal extension. YMTC helped designing and analyzing the assays to sequence *L. (L.) amazonensis *CP COOH-terminal extension and reviewed the manuscript. TCBA helped designing and performing cell culture-related assays. ALB helped designing the project, performing and analyzing T lymphocyte phenotyping assays and reviewed the manuscript. ERC helped designing, performing and analyzing *in silico *assays and reviewed the manuscript. CRA conceived the study, contributed in the design of the project, helped performing cell culture-related assays and reviewed the manuscript. All authors read and approved the final version of this manuscript.
